# Combination of surveillance tools reveals that Yellow Fever virus can remain in the same Atlantic Forest area at least for three transmission seasons

**DOI:** 10.1590/0074-02760190076

**Published:** 2019-04-29

**Authors:** Filipe Vieira Santos de Abreu, Edson Delatorre, Alexandre Araújo Cunha dos Santos, Anielly Ferreira-de-Brito, Márcia Gonçalves de Castro, Ieda Pereira Ribeiro, Nathália Dias Furtado, Waldemir Paixão Vargas, Mário Sérgio Ribeiro, Patrícia Meneguete, Myrna Cristina Bonaldo, Gonzalo Bello, Ricardo Lourenço-de-Oliveira

**Affiliations:** 1 Fundação Oswaldo Cruz-Fiocruz, Instituto Oswaldo Cruz, Laboratório de Mosquitos Transmissores de Hematozoários, Rio de Janeiro, RJ, Brasil; 2 Instituto Federal do Norte de Minas Gerais, Salinas, MG, Brasil; 3 Fundação Oswaldo Cruz-Fiocruz, Instituto Oswaldo Cruz, Laboratório de Genética Molecular de Microorganismos, Rio de Janeiro, RJ, Brasil; 4 Fundação Oswaldo Cruz-Fiocruz, Instituto Oswaldo Cruz, Laboratório de Biologia Molecular de Flavivírus, Rio de Janeiro, RJ, Brasil; 5 Fundação Oswaldo Cruz-Fiocruz, Escola Nacional de Saúde Pública, Departamento de Endemias Samuel Pessoa, Rio de Janeiro, RJ, Brasil; 6 Secretaria de Estado de Saúde, Superintendência de Vigilância Epidemiológica e Ambiental, Rio de Janeiro, RJ, Brasil; 7 Fundação Oswaldo Cruz-Fiocruz, Instituto Oswaldo Cruz, Laboratório de AIDS e Imunologia Molecular, Rio de Janeiro, RJ, Brasil

**Keywords:** Yellow Fever, amino acid changes, phylogeography, Alouatta, mosquito vectors

## Abstract

**BACKGROUND:**

In Brazil, the Yellow Fever virus (YFV) is endemic in the Amazon, from where it eventually expands into epidemic waves. Coastal south-eastern (SE) Brazil, which has been a YFV-free region for eight decades, has reported a severe sylvatic outbreak since 2016. The virus spread from the north toward the south of the Rio de Janeiro (RJ) state, causing 307 human cases with 105 deaths during the 2016-2017 and 2017-2018 transmission seasons. It is unclear, however, whether the YFV would persist in the coastal Atlantic Forest of RJ during subsequent transmission seasons.

**OBJECTIVES:**

To conduct a real-time surveillance and assess the potential persistence of YFV in the coastal Atlantic Forest of RJ during the 2018-2019 transmission season.

**METHODS:**

We combined epizootic surveillance with fast diagnostic and molecular, phylogenetic, and evolutionary analyses.

**FINDINGS:**

Using this integrative strategy, we detected the first evidence of YFV re-emergence in the third transmission season (2018-2019) in a dying howler monkey from the central region of the RJ state. The YFV detected in 2019 has the molecular signature associated with the current SE YFV outbreak and exhibited a close phylogenetic relationship with the YFV lineage that circulated in the same Atlantic Forest fragment during the past seasons. This lineage circulated along the coastal side of the Serra do Mar mountain chain, and its evolution seems to be mainly driven by genetic drift. The potential bridge vector *Aedes albopictus* was found probing on the recently dead howler monkey in the forest edge, very close to urban areas.

**MAIN CONCLUSIONS:**

Collectively, our data revealed that YFV transmission persisted at the same Atlantic Forest area for at least three consecutive transmission seasons without the need of new introductions. Our real-time surveillance strategy permitted health authorities to take preventive actions within 48 h after the detection of the sick non-human primate. The local virus persistence and the proximity of the epizootic forest to urban areas reinforces the concern with regards to the risk of re-urbanisation and seasonal re-emergence of YFV, stressing the need for continuous effective surveillance and high vaccination coverage in the SE region, particularly in RJ, an important tourist location.

Except for the rare episodes of urban Yellow Fever (YF) transmission, human infections in the Americas have been acquired from the sylvatic cycle maintained between arboreal mosquitoes and non-human primates (NHPs).^1^ Forests of northern South America have been regarded as a key territory for the maintenance and emergence of YF virus (YFV) lineages.^2^ ^,^ ^3^ In Brazil, the YFV is enzootic/endemic in the Amazon Region, which is in the north from where, it sometimes expands towards the central-west, south (S), and south-eastern (SE) regions, causing isolated epizootic events and human infections to severe epidemics of sylvatic origin.^1^ ^,^ ^2^ Epidemiological records gathered since the discovery of the YFV sylvatic cycle in Brazil in the early 1930s, as well as recent phylogenetic analyses of South American YFV samples have reinforced this concept.^1^ The epidemic character of the sylvatic YF in the extra-Amazon Brazilian territory would be a consequence of the continuous reintroductions of YFV from the endemic region in the Amazon, as the former territories could not maintain the circulation of the virus after one or two transmission seasons, due to the substantial reduction in the number of susceptible vertebrate hosts.^1^ ^,^ ^4^ ^,^ ^5^

In the last two decades, a gradual expansion of YFV has been observed towards the coast of SE and S Brazil, an area that has been considered YF-free for almost 80 years, and therefore, without vaccine recommendations.^4^ ^,^ ^6^ ^,^ ^7^ An entomological and surveillance investigation conducted in 2015-2016 to determine the composition and abundance of mosquito fauna in distinct ecosystems in the coastal SE region, particularly in the state of Rio de Janeiro (RJ) and its borders, has revealed that RJ was highly receptive and vulnerable to sylvatic YFV transmission.^8^ In late 2016 and early 2017, the virus re-emerged in SE Brazil, and the states of Minas Gerais (MG), Espírito Santo (ES), and RJ were affected in sequence, initiating the largest epidemic of sylvatic YFV ever recorded in the country.^9^ ^,^ ^10^ ^,^ ^11^ ^,^ ^12^ The affected area encompasses the biggest remnants of the Atlantic Forest in Brazil, which are often close to cities with the highest human population densities in the country, low YFV vaccination coverage and high *Aedes aegypti* infestation raising concern about the risks of YFV re-urbanisation.^4^ ^,^ ^13^ ^,^ ^14^

In March 2017, RJ reported the first human YF cases (n = 8); all of them were detected in the municipality of Casimiro de Abreu.^15^ In this first transmission season (July 2016 to June 2017), a total of 25 YFV human cases, including eight deaths, and 25 confirmed epizootics of NHPs were recorded in 17 municipalities in the northern and central regions of RJ (Fig. 1).^10^ During the second transmission season (July 2017 to June 2018), and despite vaccination efforts, 282 human YFV cases (including 97 deaths) and 71 confirmed epizootics were registered in 45 municipalities distributed across the central and southern regions of RJ.^11^ In the third cycle (July 2018 to June 2019), no human case has been recorded till date (February 2019) in RJ. Only one epizootic of NHPs in this season was confirmed by immunohistochemistry (IMH) in the southernmost municipality in RJ (Paraty), while seven other cases were diagnosed only by polymerase chain reaction (PCR) (n = 7), pending confirmation either by viral genome sequencing or IMH; some of these epizootics originated from areas without any epidemiological evidence of YFV circulation. Interestingly, all these epizootics occurred in marmosets (genus *Callithrix*) in July 2018,^16^ suggesting that they were remnants of the previous transmission season. It is noteworthy that only one and four out of the 55 municipalities affected by the outbreak recorded confirmed YFV infections in humans and NHPs, respectively, for more than one transmission season.^17^ ^,^ ^18^

Epizootic surveillance combined with rapid laboratory diagnosis (molecular biology techniques and IMH) has been considered a potential tool for the early detection of the emergence of YFV and assures quick responses; it could also protect the human population by defining the affected areas and areas at risk and intensifying vaccination drives, communication, and appropriate control measures.^6^ ^,^ ^19^ ^,^ ^20^ Simultaneous phylogeographical analyses of circulating viral genomes can also strengthen molecular epidemiology, detect the emergence of new lineages, and define transmission chains and virus dispersion routes, which in turn, may improve the timely establishment of prophylactic and control measures. Nevertheless, the efficient functioning of the epizootic surveillance depends on the support and alerts from a sensitised information network.^6^ ^,^ ^19^ ^,^ ^20^

By combining alerts from an information network constructed by us with the immediate use of the aforementioned laboratory tools, we promptly detected the re-emergence of YFV transmission in 2019 in an area where the outbreak had occurred two transmission seasons ago, and determined that virus transmission can be resilient in an Atlantic Forest zone, independent of new introductions.

## MATERIALS AND METHODS

*Sample collection* - From 2015 onwards, we have built an information network comprising several kinds of agents (e.g. residents, environmental guards, health agents, conservation unit managers, and local guides) regularly visiting or living in target areas in RJ and consisting of contact chains of key institutions and inhabitants, to continuously monitor the epizootic events of NHPs in RJ using communication technologies such as message exchange applications (FVS Abreu, unpublished data).

**Fig. 1:**
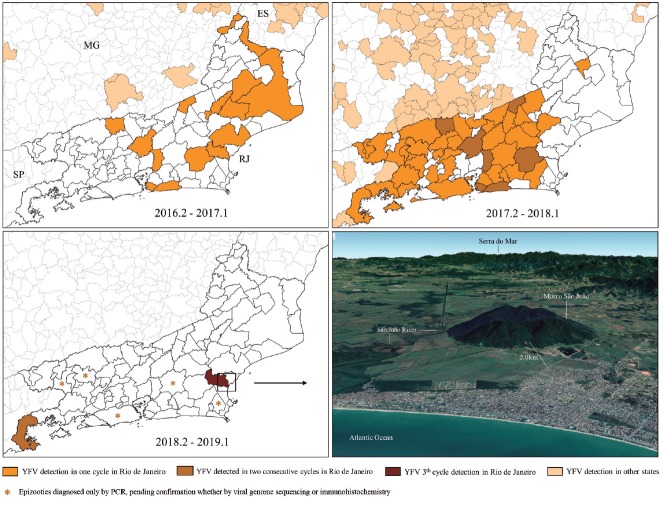
Yellow Fever virus (YFV) detection in human and/or non-human primates, per county and seasonal transmission cycle, in Rio de Janeiro and its borders. The satellite image shows Morro São João, where the howler monkey was found, and its surroundings. This woody fragment is bordered by the São João River, whose gallery forest may serve as a corridor for virus dispersion between the more continuous forests on the steps of Serra do Mar. Frontal view of Morro do São João was obtained through the Google Earth Software, accessed on 22th Feb 2019.

On January 9th 2019, we received an alert from the information network reporting a dying howler monkey in Morro de São João (22º32’37.98”S 42º0’43.88”W), a mountain with a dense vegetation cover located in the coastal lowlands of Casimiro de Abreu, the municipality where the first human YF case was reported in RJ and whose last detected YFV circulation was recorded in early 2017. The animal was found only 2.0 km away from a considerably dense urban area (Fig. 1).

**Fig. 2:**
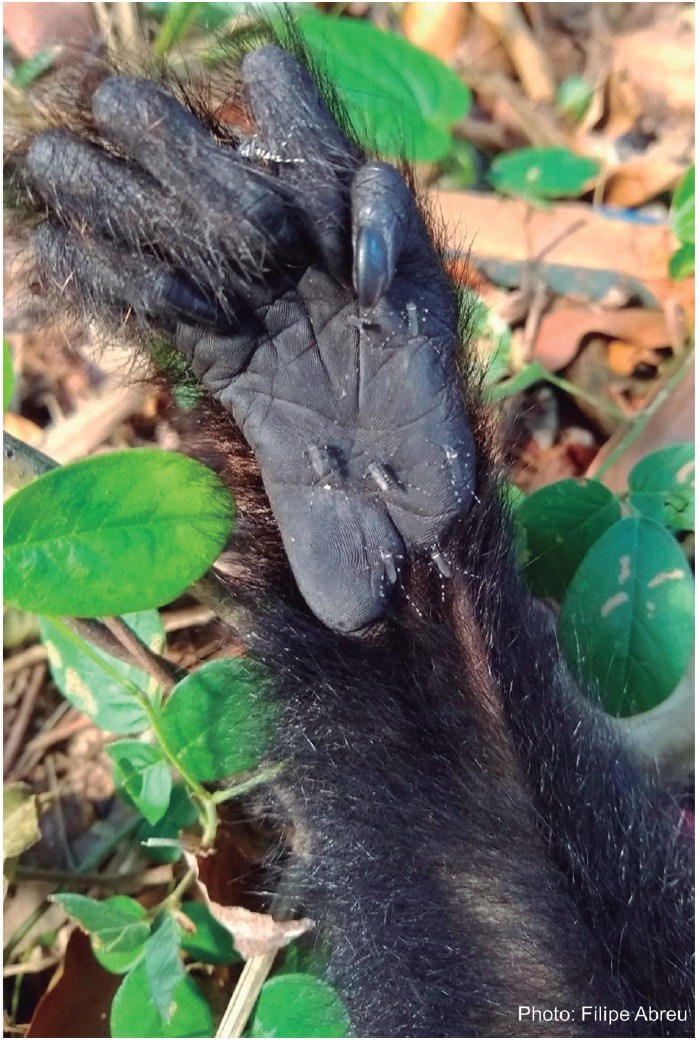
*Aedes albopictus* mosquitoes trying to bite one hand of the recently dead *Alouatta guariba clamitans* found in Morro de São João (22º32’37.98”S 42º0’43.88”W) on January 10th, 2019.

Our team immediately went to the area (166 km from the lab). On our arrival, the monkey had already died. We were attacked by numerous *Aedes albopictus* mosquitoes, which also tried to bite the recently dead animal, apparently without success, as engorged females were not observed (Fig. 2). We performed the necropsy and sample collection on the field, in accordance with the biosafety protocols and the current biodiversity laws.^20^ The animal, a young adult female *Alouatta guariba clamitans* without any wound or external mark, was named RJ155. Samples of the animal’s viscera (liver, spleen, heart, kidney, lung), whole blood, and serum were immediately frozen on dry ice. The carcass was kept refrigerated until delivery at the state health department of RJ, with the epizootic notification form filled out by the municipal health division, as officially recommended. One week later, we visited the site again for mosquito collection. Mosquitoes were collected and tested in pools from 1 to 10 individuals, according to the species, as previously described.^8^ ^,^ ^21^

*RNA extraction, virus detection, and sequencing* - On the same day, RNA was extracted from 140 μL of RJ155’s serum and whole blood using the Qiagen RNA Viral Kit, following the manufacturer’s recommendations. Real-time reverse transcription quantitative PCR (RT-qPCR) was performed in duplicate, as described previously.^8^ ^,^ ^21^ The set of primers utilised for the PCR and viral genome sequencing procedures followed a previous report.^9^ The nucleotide sequence was determined by capillary electrophoresis at the sequencing facility of Fiocruz-RJ (RPT01A ― Sequenciamento de DNA - RJ). The sequences were assembled using SeqMan Pro version 8.1.5 (DNASTAR, Madison, WI, USA). The amino acid differences, as well as nucleotide and amino acid distances, were calculated using the molecular evolutionary genetics analysis (MEGA) 7.0 program.

*YFV dataset assembly* - The complete polyprotein open reading frame [complete coding sequence (CDS), 10,239 nt in length] of the genome of the newly generated 2019 YFV from the Casimiro de Abreu municipality was combined with all American YFV sequences available in GenBank on January 2019 (www.ncbi.nlm.nih.gov/genbank/) with known dates and countries of collection, covering at least 99% of the viral CDS, and with an associated publication. The sequences were aligned using MAFFT^22^ and classified into South American genotypes I and II by reconstructing a maximum likelihood (ML) phylogenetic tree with PhyML^23^, under the best substitution model selected by jModelTest v1.6.^24^ Branch support was estimated with the approximate likelihood-ratio test (aLRT). Only YFV sequences classified as South American I genotype (SA-I) were retained for further analyses.

*Evolutionary and phylogeographic analysis* - The spatiotemporal viral diffusions were reconstructed by Bayesian inference with Markov chain Monte Carlo (MCMC) sampling, as implemented in the BEAST v1.8.4 package,^25^ using BEAGLE^26^ to improve the running time. The GTR+I nucleotide substitution model selected by jModelTest v1.6,^24^ a relaxed lognormal molecular clock model calibrated with a normal prior based on previous estimates,^9^ and the non-parametric Bayesian skyline coalescent model were used in case of all Bayesian phylogeographic inferences. The best discrete (symmetric or asymmetric)^27^ and continuous (homogenous or heterogeneous)^28^ phylogeographic models were selected using a marginal likelihood estimator (MLE), employing the path sampling (PS) and stepping-stone sampling (SS) approaches.^29^ The analyses were run for 10^8^ MCMC iterations, and the convergence (effective sample size > 200) was assessed using TRACER v1.7 (beast.community/tracer) after discarding a 10% burn-in. The maximum clade credibility (MCC) trees were summarised using TreeAnnotator v.1.8.4 and visualised with FigTree v.1.4.4 (tree.bio.ed.ac.uk). The viral spatiotemporal diffusion was analysed and visualised in SPREAD,^30^ and further projected in maps generated using the QGIS software (qgis.org) including cartographic information provided by the Brazilian Institute of Geography and Statistics (https://mapas.ibge.gov.br/) and information about the Atlantic Forest remnants (2016 estimates) from the Brazilian National Institute for Space Research and SOS Mata Atlântica Foundation (mapas.sosma.org.br/).

*Genetic distance and selection analysis* - The ancestral CDS of the node encompassing all YFV sequences from RJ closely related to the 2019 YFV strain was reconstructed by Bayesian inference as described above, and computed using the Geneious 9.1.4 program (https://www.geneious.com). Genetic differences relative to the inferred ancestral CDS were calculated based on the global (*d*_*nt*_ ) (TN model), synonymous (*dS*) and nonsynonymous (*dN*)^31^ ^,^ ^32^ nucleotide distances, which were obtained using MEGA 7. The analysis of selection was performed on the Datamonkey web server (www.datamonkey.org) using the HyPhy package^33^ with the fixed-effect likelihood (FEL)^33^ and mixed effect model evolution (MEME)^34^ methods, and incorporating the best nucleotide substitution model.

*Ethics* - Our methods and protocols were previously approved by the institutional Ethics Committee for Animal Experimentation (protocol CEUA/IOC-029/2016, license L-037/2016), the Brazilian Ministry of the Environment (SISBIO 41837-3 and 54707-4), and RJ’s Environment agency (INEA 012/2016 and 019/2018).

## RESULTS

RT-qPCR analysis detected YFV RNA in the serum and whole blood collected from RJ155 (mean of CT value = 19.7); this diagnosis was further confirmed by sequencing the complete genome of YFV (GenBank accession number: MK533792). However, the 278 mosquitoes (one *Haemagogus janthinomys*, two *Hg. leucocelaenus*, five *Sabethes albiprivus*, 15 *Ae. albopictus*, and 255 *Ae. scapularis*) collected at the Morro de São João a small-time interval after the notification alert for the dying monkey tested negative for YFV.

The identity between the RJ155 YFV and the YFV sequenced in the 2016-2017 transmission season from the neighbouring counties is high, from 99.8 to 99.9% and from 99.7 to 100%, which are the nucleotide and amino acid identity values, respectively [Supplementary data (Table I)]. The number of nucleotide changes indicates the evolution of the viral genome, most probably due to a bottleneck effect, promoted by the dissemination of the virus into a small number of hosts while circulating silently in the zone [Supplementary data (Table II)]. However, we noticed six amino acid alterations not only in the YFV samples from bordering municipalities, but also in case of all YFV genomes described so far in the ongoing outbreak (Table). The amino acid variations mainly map at the following non-structural YFV proteins: C 103 (R); NS1 51 (D); NS3 (G); NS4A (I); NS5 391 (K), NS5 622 (M), and NS5 645 (I).

**TABLE:** Polyprotein polymorphisms present in the 2019 Yellow Fever virus (YFV) from Morro de São João in comparison with previous circulating YFV in Casimiro de Abreu, and in the bordering municipalities (Macaé and Silva Jardim) in the Rio de Janeiro state

YFV sample / polyprotein position	103	829	1744	2897	3128	3151
MK533792/2019/RJ155/MorroSJoão/M	R	D	G	K	M	I
MF423375/2017/RJ87/MacaéAtalaia/M	Q	E	E	N	I	T
MF538786/2017/RJ104/Guapimirim/M	Q	E	E	N	I	T
MF423376/ 2017/RJ94/MacaéSana/M	Q	E	E	N	I	T
MF434851/ 2017/H199/SilvaJardim/H	Q	E	E	N	I	T

**Fig. 3:**
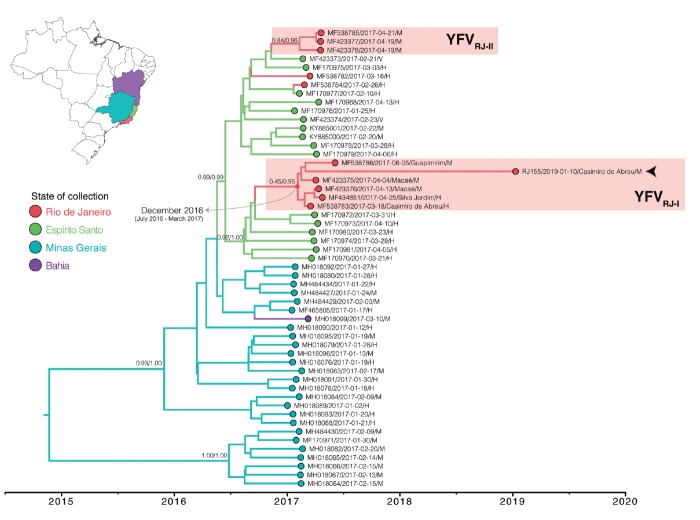
maximum clade credibility phylogeographic tree of the Yellow Fever virus (YFV) strains involved in the ongoing outbreak. The colours’ branches represent the most probable location of their descendent nodes, as indicated in the legend and the map. The posterior and posterior state probability (PP/PSP, respectively) of key nodes are indicated above the branches. All horizontal branch lengths are drawn to a scale of years. The two lineages found in Rio de Janeiro state (YFV_RJ-I_ and YFV_RJ-II_) are indicated by red shaded boxes, and the RJ155 samples are indicated by arrows.

The ML phylogenetic analysis revealed that the RJ155 YFV 2019 genome showed the highest support values (aLRT = 1) when clustered with other YFV strains from the ongoing outbreak in SE Brazil [Supplementary data (Figure)]. In order to better understand the geographic origin of the RJ155 YFV, we performed a discrete Bayesian phylogeographic analysis of all YFVs from the current SE Brazilian outbreak. As there was no significant evidence favouring one of the discrete phylogeographic models [Supplementary data (Table III)], we have presented the results of the symmetric one. The inferred Bayesian MCC tree supports at least four independent introductions of YFV into RJ from ES and confirmed at least two viral transmission chains in RJ^9^ (Fig. 3). The RJ155 YFV clustered within the RJ transmission chain here called YFV_RJ-I_ that likely originated in RJ [posterior state probability (PSP) = 0.95] around December 2016 [95% Bayesian credible interval (BCI) July 2016-March 2017]. The YFV_RJ-I_ clade also comprises an older YFV sample from Casimiro de Abreu (March 2017) obtained from a human case and other YFVs obtained from humans and NHPs in neighbouring municipalities (Macaé, Silva Jardim and Guapimirim) from April to June 2017.

**Fig. 4:**
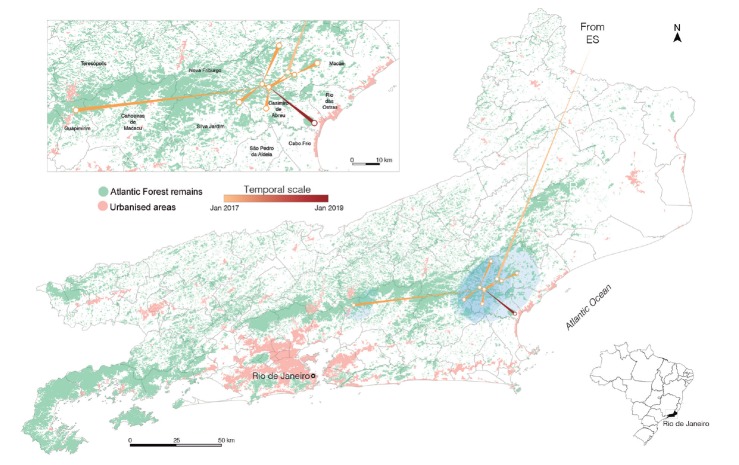
reconstructed spatiotemporal diffusion of the Yellow Fever virus (YFV)_RJ-I_ lineage. The branches of the YFV_RJ-I_ lineage phylogeny were arranged in space (map of Rio de Janeiro state with municipalities borders) according the locations of known (external) and inferred (internal) nodes (circles). The blue shaded regions represent the 95% credible regions of the inferred internal nodes. The branch thickness represents the spread direction (thin to thick) between locations. The inset shows a close view of viral dissemination, with the municipalities’ names. ES: Espírito Santo state.

To assess the spatial spread of the YFV_RJ-I_ lineage with more precision, we employed a relaxed random walk phylogeographic model with a lognormal distribution, selected as the fittest continuous phylogeographic model [Supplementary data (Table IV)]. The continuous diffusion inference placed the origin of the YFV_RJ-I_ lineage in the municipality of Macaé in January 2017 (BCI September 2016-February 2017), from where it rapidly spread throughout the coastal steps of the Serra do Mar mountain chain and the lowlands shared with the neighbouring municipalities of Casimiro de Abreu and Silva Jardim, and the more distant municipality of Guapimirim (Fig. 4). The continuous diffusion model supports that the dissemination range of the YFV_RJ-I_ lineage covers an extensive area of Atlantic Forest remains situated along the coastal side of Serra do Mar, shared by the municipalities of Macaé, Rio das Ostras, Casimiro de Abreu, Nova Friburgo, and Silva Jardim. This zone is very close to urbanised areas, including the place where the RJ155 was sampled, which is located only two kilometres away from the contiguous urban perimeter of Casimiro de Abreu and Rio das Ostras (Fig. 1). These results clearly support that the YFV has persisted in the Atlantic Forest area of RJ for three consecutive YFV transmission seasons (2016-2017, 2017-2018, and 2018-2019).

**Fig. 5:**
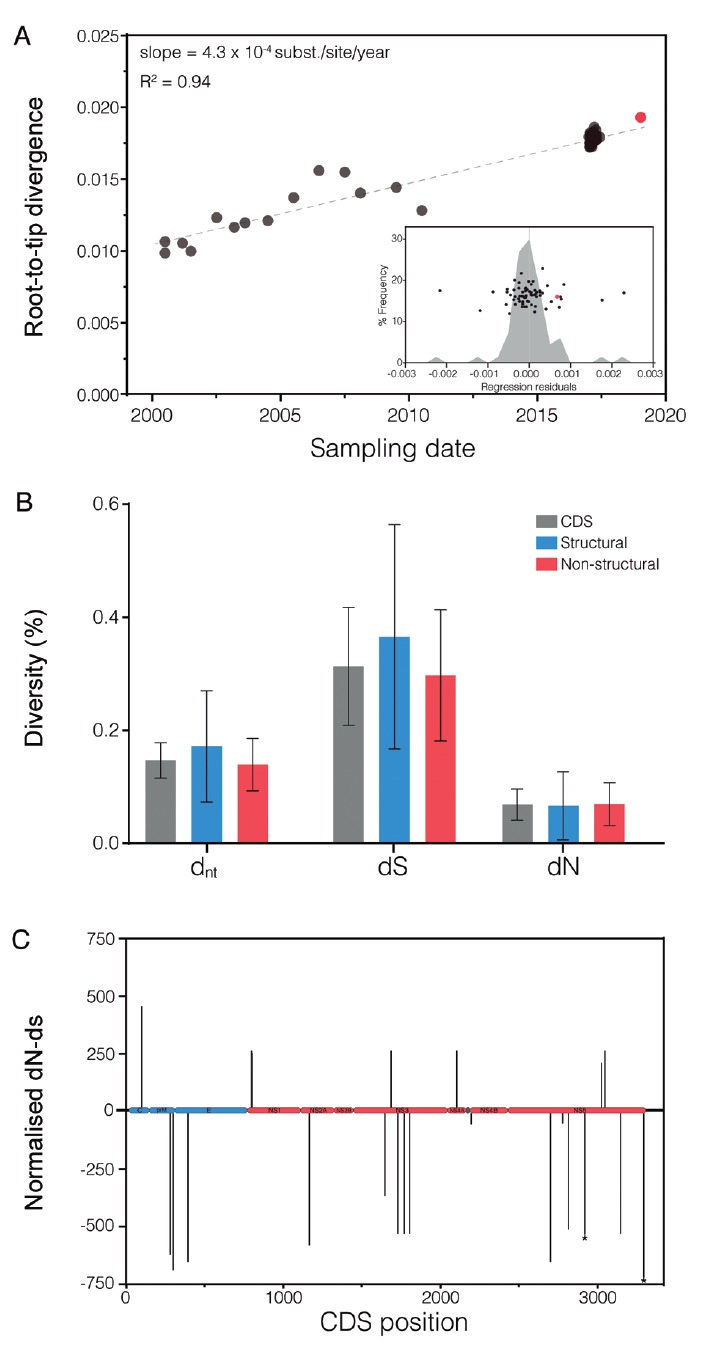
Yellow Fever virus (YFV)_RJ-I_ divergence and selection analysis. (A) Root-to-tip regression of sequence sampling date against genetic divergence from the root of the South American I genotype. The inset panel contains a histogram and scatterplot of the residuals of the linear regression. The RJ155 sequence is represented using red colour. (B) Mean divergence of RJ155 at the nucleotide (*d*_*nt*_ ), synonymous (*dS*), and non-synonymous (*dN*) levels in the complete coding sequence (CDS, grey), and the structural (blue) and non-structural (red) genes. The columns represent mean distances, and error bars represent ± standard errors of the mean obtained by bootstrap. (C) Normalised *dN-dS* values by codon position across the complete YFV CDS (structural and non-structural genes are coloured blue and red, respectively). The asterisks indicate positions with p-values < 0.1 in the REL analysis.

To examine the genetic divergence of the RJ155 YFV relative to previous virus samples, we plotted the root-to-tip divergence from the ML tree as a function of sampling time (Fig. 5A). We found a strong correlation between the divergence and time of sampling (R^2^ = 0.94). The RJ155 YFV showed a low deviation from the mean regression line, with residuals comparable with other YFV genomes sampled previously. The RJ155 showed a low mean global nucleotide genetic divergence (*d*_*nt*_ > 0.2%) from the ancestral CDS of the YFV_RJ-I_ lineage. The mean nucleotide synonymous (*dS*) distance was much higher than the mean nonsynonymous (*dN*) distance in both the structural and non-structural genomic regions (Fig. 5B). The analysis of selective pressure (normalised *dN-dS* along the CDS) showed an overrepresentation of sites accumulating synonymous substitutions. No positively selected codon positions were identified at the entire CDS sequence by the MEME or REL methods, and two sites in the NS5 gene (positions 3017 and 3406) displayed a trend (p-values 0.081 and 0.072, respectively) towards negative selection (Fig. 5C). These observations support the claim that the evolution of the YFV_RJ-I_ lineage was mainly driven by genetic drift.

## DISCUSSION

The present work describes the first record of YFV circulation in RJ in 2019, which essentially consists of the primary sign of virus re-emergence in the state since July 2018, when the epidemiological transmission season had started. Epizootics reported in marmosets in the central and southern zones of RJ nearly six months ago were remnants of the previous transmission season. In turn, we report a YFV infection in a howler monkey from the north-coastal zone, where the YF outbreaks and epizootic records had peaked in early 2017 and then moved southward. This suggests that viral circulation may be soundless in the coastal SE Brazil. Moreover, our phylogeographic and evolutionary analyses showed that YFV may persist in this zone for at least three consecutive transmission seasons, without the need for new introduction. These data unprecedentedly demonstrate that YFV can be locally maintained in the Atlantic Forest for more than two transmission seasons.

When investigating YF sylvatic epidemics occurring in SE Brazil during the 1930s and 1940s, the authors concluded that in this region, YFV spread in the form of an intermittent wave from infected areas to unaffected neighbouring sites, involving a series of annual outbreaks coinciding with the rainy season (summer), and essentially stopping during the winter, only to retake its course and spread in the next rainy season into sites that were close to those affected during the previous summer.^5^ ^,^ ^35^ ^,^ ^36^ Despite describing the same phenomenon in the current outbreak, Rezende et al.^37^ named this spreading as persistence. Here, we demonstrated that besides spreading to new areas, YFV was able to persist in the same zone for at least three transmission seasons in RJ. The mosaic pattern of the Atlantic Forest may have contributed to the maintenance of YFV circulation in such a zone for at least three seasons, where some isolated fragments may not be affected by the expanding wave. Such “virgin islands,” where the YFV has not circulated, may serve as new focal points for viral re-emergence and amplification.

Besides the fragmentation of the Atlantic Forest, the increase of NHP diversity and abundance may have also contributed to YFV persistence in RJ. Previous studies have concluded that YFV typically remains during only one, but not for more than two transmission seasons in the same area in the Atlantic Forest of the SE region.^5^ ^,^ ^35^ ^,^ ^38^ ^,^ ^39^ ^,^ ^40^ Taylor and Fonseca-Cunha,^36^ while describing the 1934-1936 sylvatic YFV epidemic in the SE, claimed that “the virus appeared to “burn itself out” as it progressed and rarely lasted longer than one season in any given locality”. The main reason for this pattern would be the reduction in the number of local susceptible hosts due to the rapid and intense transmission and spatial spread of YFV^39^ ^,^ ^40^ across the fragmented Atlantic Forest in the SE region, the most populated and developed Brazilian region. On the other hand, in the Amazon Region, the dense and continuous forests, together with the great diversity of NHPs, enable an almost perennial circulation of the virus. In fact, YFV had disappeared spontaneously from the coastal Atlantic Forest for almost 80 years. However, during the last decades, environmental and biodiversity protection policies have achieved considerable success. Significant augmentation of conservation units and numerous reforestation initiatives, and the recovery of ecological corridors in the Atlantic Forest have been observed; this may have expanded the zones with suitable environmental conditions to support higher mosquito and NHP species diversity and abundance, especially compared to the case for when the Brazilian developmental policies were put into practice after the second war.^1^ ^,^ ^41^ Interestingly, the zone where we detected the virus during the third transmission season (2018-2019) coincides with the RJ Atlantic Forest region which has one of the greatest diversities of native NHP genera, including species of *Alouatta*, *Sapajus* (capuchins), *Callicebus* (titis), *Callithrix* and *Leontopithecus* (lion-tamarins); the endangered species *L. rosalia* (golden-lion-tamarin), which was affected during the 2017-2018 season,^34^ is endemic to this zone of RJ, and the major conservation unit established for the protection of this species is in the same zone. Two recent studies that modelled the epidemiology of YF found that the distribution and diversity of NHP genera are highly associated with the risk of YFV outbreak.^42^ ^,^ ^43^

YFV was also likely maintained in RJ through vertical transmission in the vectors between seasonal peaks of disease spread,^44^ which in Brazil occurs during the summer, when higher mean temperatures and abundant rainfall favour the reproduction of mosquitoes like *Haemagogus sp*., a tree-hole breeding species.^45^ ^,^ ^46^ Although we have not found infected mosquitoes in our collections made where we sampled the RJ155 YFV, primary (*Hg. leucocelaenus*, *Hg. janthinomys*) and secondary or potential (*Sa. albiprivus*, *Ae. scapularis*, *Ae. albopictus*) vectors^8^ ^,^ ^47^ ^,^ ^48^ were detected. The most abundant mosquito species, i.e. *Ae. scapularis* and *Ae. albopictus*, usually bite at the ground level, which is a behaviour limiting their chance of getting infected by feeding on viraemic forest canopy-inhabiting NHPs such as howler monkeys. However, many sick monkeys get down from the canopy and agonise for several hours on the ground; this is when they may be easily bitten by such species of mosquitoes.^8^ *Ae. albopictus* is considered a probable bridge vector for YFV at the urban area-forest interface in Brazil;^47^ ^,^ ^49^ its contact with viraemic howler monkeys inhabiting regions that are in close proximity to an urban area registered in our study (Figs 1-2) has been highlighted as an important risk factor for YF re-urbanisation in the SE.^1^

YFV evolution in fragmented habitats through vertical transmission in the vectors might impose strong bottlenecks to viral diversity. The divergence accumulated in the RJ155 YFV, however, was compatible with the expected mean divergence for YFV, indicating that the substitutions were not accumulated in this strain at a faster rate compared with that in other viral strains. The overrepresentation of negative dN-dS values found in the YFVRJ-I lineage indicates that the vast majority of mutations in these viruses are synonymous and fixed by purifying the selection and/or genetic drift, as previously observed for YFV.^50^ Arthropod-borne viruses like YFV are characterised by higher levels of negative selection pressure than RNA viruses transmitted by other routes, which is probably a consequence of a life cycle involving phylogenetically divergent hosts.^51^

Our study clearly supports that YFV circulation may be soundless in the coastal SE and that the transmission of this virus may gain force, and cause its re-emergence in the form of epizootics when conditions are suitable. Silent viral circulation or absence of notifications can lead to the demobilisation and deintensification of prophylactic and communication actions, which can have serious consequences. The local persistence of YFV and the possibility of seasonal re-emergences reinforce the need for maintaining continuous surveillance and high vaccination coverage in the SE, particularly in RJ, a state that receives a large influx of (vaccinated and unvaccinated) tourists during the summer who could become infected and export the virus to other states and countries.^52^ Effective epizootic surveillance, combined with fast diagnostic and phylogenetic analyses is critical in raising timely awareness and establishing control measures. Using this strategy, we could alert the state and municipal health systems in RJ about YFV re-emergence within 48 hours allowing the authorities to take appropriate preventive actions immediately.
