# Supplementary material for: Combination of surveillance tools reveals that Yellow Fever virus can remain in the same Atlantic Forest area at least for three transmission seasons
Source: Mem Inst Oswaldo Cruz. 2019 Apr 29;114:e190076. doi: 10.1590/0074-02760190076 (PMC6489371; doi:10.1590/0074-02760190076)
Supplement: Supplementary file 1 [file 1678-8060-mioc-114-e190076-s.pdf]

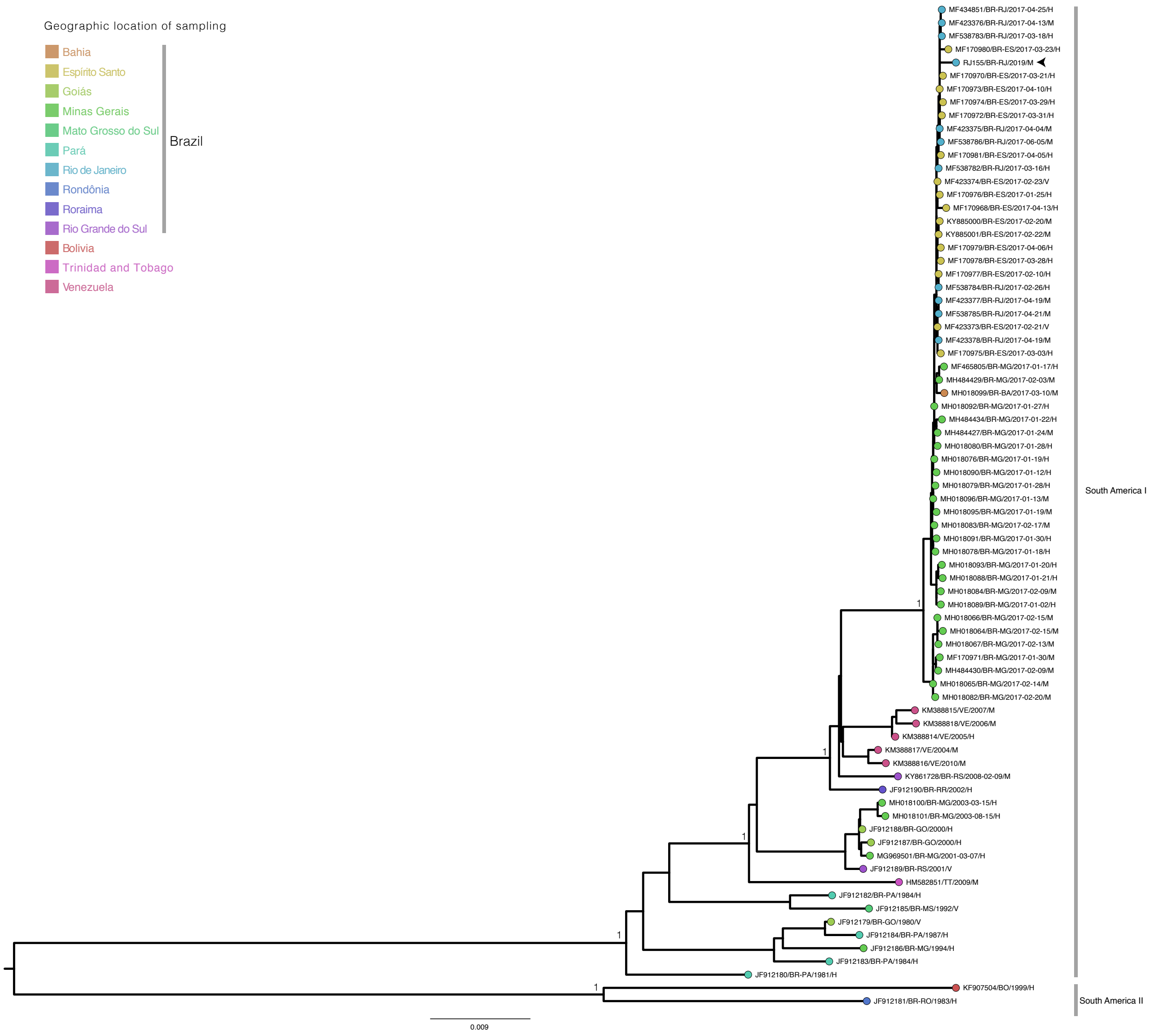

Maximum likelihood phylogeny of the complete coding sequences of the South American I and II genotypes of Yellow Fever virus (YFV). The tip circles are coloured following the legend at the top left, indicating the country or Brazilian state where the sample was collected. The branch lengths are drawn to scale, with the bar at the bottom indicating the nucleotide substitutions per site; the aLRT support values of key nodes are also indicated. The grey bars highlight each genotype clade. The RJ155 sample is indicated with an arrow.

TABLE I

Identity matrix of the Yellow Fever virus (YFV) complete coding sequence (CDS) from Casimiro de Abreu and in the bordering municipalities (Macaé and Silva Jardim). The nucleotide identity values are highlighted in grey and the amino acid identity values in green

YFV strains	1 % of identity (ID)	2	3	4	5	6
1. MK533792/2019/RJ155/MorroSJoão/M	ID	99.8	99.8	99.8	99.8	99.8
2. MF423375/2017/RJ87/MacaéAtalaia/M	99.7	ID	99.9	99.9	99.9	99.9
3. MF538786/2017/RJ104/Guapimirim/M	99.7	100	ID	99.9	99.9	99.9
4. MF423376/2017/RJ94/MacaéSana/M	99.7	100	100	ID	99.9	99.9
5. MF434851/2017/H199/SilvaJardim/H	99.7	99.9	99.9	99.9	ID	99.9
6. MF538783/2017/H191/CasimiroAbreu/H	99.7	99.9	99.9	99.9	99.9	ID

CC BY

TABLE II

Genome variations between the complete coding sequence (CDS) from 2019 Yellow Fever virus (YFV) (RJ155) and 2017 YFV, which circulated in Casimiro de Abreu and the bordering municipalities (Macaé and Silva Jardim)

YFV samples / nucleotide position	308	879	936	1224	2476	2487	3621	5113	5231	5361	5496	5595	6526	8376	8691	8733	9051	9384	9452	9765	10218
MK533792/2019/RJ155/MorroSJoão/M	G	T	A	G	G	T	T	T	G	T	C	T	A	G	A	A	C	G	T	C	G
MF423375/2017/MacaéAtalaia/M	A	C	G	A		G	C		A	C	T	C		A	C	C		T	C	T	
MF538786/2017/Guapimirim/M	A	C	G	A		G	C	C	A	C	T	C		A	C	C		T	C	T	
MF423376/2017/MacaéSana/M	A	C	G	A		G	C		A	C	T	C		A	C	C	T	T	C	T	A
MF434851/ 2017/ SilvaJardim/H	A	C	G	A	T	G	C		A	C	T	C		A	C	C		T	C	T	A
MF538783/2017/CasimiroAbreu/H	A	C	G	A		G	C		A	C	T	C	G	A	C	C		T	C	T	A

TABLE III

Comparison of discrete spatial models fit to the 2017-2018 Brazilian Southeast Yellow Fever virus (YFV) dataset

Model	PS Log ML[a]	Models compared	Log BF[b]	SS Log ML	Models compared	Log BF
Sym	-15998	-	-	-15998	-	-
Asym	-15998	Sym/Asym	0	-15998	Sym/Asym	0

a: log marginal likelihood (ML) estimates for the different continuous phylogeographic models obtained using the path sampling (PS) and stepping-stone sampling (SS) methods. *b*: the Log Bayes factor (BF) is the difference of the Log ML between of alternative (H1) and null (H0) models (H1/H0). Log BF > 3 indicates that model H1 is more strongly supported by the data than model H0.

TABLE IV

Comparison of continuous spatial models fit to the 2017-2018 Brazilian Southeast Yellow Fever virus (YFV) dataset

	Homogeneous Brownian diffusion	Heterogeneous RRW Cauchy	Heterogeneous RRW Gamma	Heterogeneous RRW Log-normal
PS - Log ML[a]	-16120	-16095	-16088	**-15679**
PS - Log BF[b]	441	416	409	Fittest model
SS - Log ML[a]	-16120	-16096	-16088	**-12549**
SS - Log BF[b]	3571	3547	3539	Fittest model

a: log marginal likelihood (ML) estimates for the different continuous phylogeographic models obtained using the path sampling (PS) and stepping-stone sampling (SS) methods. *b*: the Log Bayes factor (BF) is the difference of the Log ML between of alternative (H1) and null (H0) models (H1/H0). Log BF > 3 indicates that model H1 is more strongly supported by the data than model H0. RRW: relaxed random walk.
